# The Medical Association of Georgia

**Published:** 1885-05

**Authors:** 


### THE MEDICAL ASSOCIATION OF GEORGIA.

The Medical Association of Georgia held its thirty-sixth annual session in Savannah, April 15th, 16th and 17th.

There were many valuable papers and reports presented, which will appear in THE JOURNAL from time to time during the year.

We are pleased to state that the Association, by a unanimous vote, adopted THE JOURNAL as the Journal of the Association, and that the minutes, papers, reports, etc., will be published regularly as the papers of the American Medical Association are now published in the Journal of that Association. In future the Transactions will not be published in book form as heretofore.

This new arrangement will prove beneficial to the members in more ways than one. Each member of the Association who pays his due will be furnished with THE JOURNAL *free* for one year, beginning with the present issue.

The papers of the members will have, under this new arrangement, a much larger circulation than they ever had before. Where hundreds read them in book form, thousands will read them now. The expenses of the Association will be reduced about one-half, and the membership, we have no doubt, will be very greatly increased.

It is our desire to make THE JOURNAL as attractive and useful as possible, and to that end we invite the members of the Association and the Profession generally to send us short reports of cases, and to furnish us any item of interest to the Association that may occur in their several localities.

### ITEMS.

MEMBERS of the Medical Association of Georgia will find on advertising page 19 an important notice from the Secretary.

DR. JAMES LAWRENCE LITTLE, Professor of Surgery in the New York Post-Graduate School, died on April 4, from peritonitis.

THE *Public Herald*, of Philadelphia, edited by Lum Smith, Esq., is waging a furious crusade against frauds of all kinds. Let the good work go on.

DR. T. O. POWELL, Superintendent of the Insane Asylum at Milledgeville, gave us a call on his way to New Orleans, where he goes as a delegate to the American Medical Association.

CORRECTION.—On page 172, first line, for "Nokitansky" read "*Rokitansky;*" ninth line for "ten" per cent, read "*two*" per cent. Page 173, fourth line, for "brought" a cure, read "*wrought*" a cure.

DR. E. H. RICHARDSON, of Cedartown, one of the leading physicians of his section, passed through Atlanta on Sunday, April 26, on his way to the meeting of the American Medical Association in New Orleans.

WE have received from the *Illustrated Medical Journal* Co., Detroit, Mich., several sets of their *perforated adhesive Journal labels*. The lists include Journals of Medicine, Pharmacy and Hygiene, both in the United States and Canada, and are very useful.

PREVALENCE OF CANCER.—Forty years ago, Mr. Thomas Wilkinson King drew the following results from some one thousand *post mortem* examinations performed at Guy's Hospital: "Of all females who die above forty-four years of age, nearly one-half have cancers; of males, one-eighth. Of males above sixty-five, one-fifth of all who die are cancerous.—*N. Y. Med. Record.*

DR. J. B. ROBERTS, of Sandersville, first Vice-President of the State Medical Association, was married in this city on Sunday, April 26, to a daughter of Mr. Green B. Roberts.

DR. J. W. HOLLINGSWORTH, of Luna Landing, Ark., writes: "Will some of the many readers of THE JOURNAL give through its columns the most approved treatment of swamp fever?"

THE Pennsylvania Legislature, following the lead of several other States, has passed a bill ordering instruction in anatomy and physiology, with special reference to the use of stimulants, to be made in the public schools of the State.—*Polyclinic.*

AT the recent meeting of the Medical Society of the State of Tennessee, held in Nashville, Dr. Thos. L. Maddin, of Nashville, was elected President; Dr. C. C. Fite, Nashville, Secretary; and Dr. Deering J. Roberts, of Nashville, Treasurer.

"CONGESTION" has made its appearance in a convict camp in Wrightsville. A convict died there suddenly a few days since, and the verdict of the coroner's jury was that death resulted from congestion. This fatal malady seems to be known only among coroners.

AN IMPOSTOR.—A man calling himself Dr. Edward Fishblatt is evidently imposing upon the people of Wisconsin, as the following letter will show:

GREEN BAY, WIS., March 25, 1885.

POSTMASTER, ATLANTA—*Dear Sir:* Will you kindly inform me whether there is an institution in your city known as the Atlanta Medical College, and whether Dr. Edward Fishblatt, of Janesville, Wis., was formerly a Professor therein? He so advertises himself here. If there is not now such an institution, please tell me whether there was formerly.

This will oblige yours truly, D. I. FOLLETT.

We have had several inquiries in regard to this fellow, and have invariably answered, "We know no such person. He was never connected with the Atlanta Medical College in any way."

TREATMENT OF CANCER.—A correspondent of the *British Medical Journal* advocates treating small cancerous tumors by arsenical paste, after a preliminary hypodermic injection of cocaine. He reports one case where this plan appears to have been efficacious.—*Medical Review.*

DEATH UNDER CHLOROFORM.—A man died recently in England, under the influence of chloroform-vapor, which had been administered to him for the performance of an operation. An inquest was held, and a verdict was returned of "death from chloroform, properly administered."

HOW TO PREDICT SEX.—Dr. S. T. Lowry diagnosticates the sex of the fœtus, at or near full term, by the pulse rate. He says that a pulse recording over 135 beats to the minute will prove to be that of a female child, while, on the other hand, if it be below that number, the child is a male.—*Texas Courier-Record.*

WM. WOOD & CO. announce an index to the volumes of their medical library, to include all the volumes published to the end of 1885. It will appear at the end of this year. As the series will then contain eighty-four volumes, the index will be a large book. It will be sold separately to such as desire it.

IN some cases of inebriety a most remarkable exaltation of memory is apparent, which begins after the use of alcohol and grows very intense for a long time, then gradually fades away. Sometimes these memories concern events which have been unpleasant, and make vivid the wrongs, real or imaginary; or they are of a more pleasing nature, in which the happy events of life are lived over with great intensity. In one case a patient gives this as a reason for the use of alcohol, by which he could live over the golden hours of the past. These changes of memory are of great diagnostic interest, and should be studied in every case.—*Journal of Inebriety.*

WE are pained to learn of the serious illness of the venerable Dr. D. B. Searcy, of Bolingbroke. He is one of the few physicians living who was present at the organization of the Medical Association of Georgia in Macon in 1849. We trust that he may soon be restored to health.

THERE is a proposition before the Association to locate it in one of the larger cities. A committee was appointed at the recent meeting to consider the advisability of the measure, and report at the meeting in Augusta next year. It is a matter that ought to be fully discussed before action is had. THE JOURNAL is open to the members for expressions of opinions regarding the change.

IOWA is the illiterate physician's paradise, no requirement being made for the practice of medicine. If a man or woman has the gift of gab, lots of cheek, especially if he or she can dress well and make a good appearance, they will succeed in making a practice. The less education he has, the better. Little or no moral character required, either. This is the status of the medical profession in Iowa.—*Iowa State Med. Journal.*

SOLUBLE ELASTIC CAPSULES OF QUININE SULPHATE.—Pharmacy is yearly establishing more and more firmly its right to the position of handmaid to medicine, and the days of crude drugs and nauseous preparations in prescriptions are about numbered. Among the latest devices which it has placed in the hands of physicians are the Soluble Elastic Capsules of Quinine Sulphate placed on the market by that enterprising firm, Messrs. Park, Davis & Co., of Detroit. These capsules, besides furnishing the completest possible disguise to the salt, offer not the slightest obstacle to their deglutition. They possess the additional feature of containing the exact quantity of the drug specified on their labels, their contents being separately weighed before being encapsuled. The objections to the exhibitions of quinine, even in the case of the most fastidious, are thus completely removed.

A DRUGGIST in New York by his carelessness has got into trouble. He gave, by mistake, Dovers powder to an infant that had been prescribed for some one else. Not long after the mother of the child had administered the first powder, the druggist came to her house and told her that he had given her the wrong medicine. He advised her to keep the child awake and give it strong coffee, but asserted that there was nothing dangerous about the powders. The next morning, however, the child was dead. An inquest was held, and the druggist held in $1,000 bail.

IT is a fact not well understood that inebriates, under the influences of spirits, are especially prone to heat, apoplexy, and cerebral congestion. In most cases it may be safely assumed that sunstroke is provoked by the use of alcohol, and where a case is found in the streets suffering from *coup de soleil*, he is both a neurotic and alcoholic. Hence the use of alcohol, where the person is exposed to the sun, is a source of great danger.—*Dr. Christie in Journal of Inebriety*.

THE following story is told of a physician of Dayton, O.: The doctor was recently attending a case of labor in the family of one of his patrons, who, though a very excellent man, is a little slow in the payment of his medical bills. Immediately after the birth of the child, the father nervously asked: "Doctor, is the baby marked?" "Yes," quietly remarked the doctor, "it is marked 'C. O. D!'" The bill for that baby was promptly settled.—*Ex.*

THERE will be issued by the New England Publishing Co., Sandy Hook, Conn., during the month of May, a book entitled BERLIN AS A MEDICAL CENTRE, by Horatio R. Bigelow, M. D., of Washington, D. C. This book will be a complete and accurate medical guide to Berlin, giving instructions in reference to board, clinics, lectures, expenses, etc., and all information that will be necessary for the medical student abroad. The price will be $2.00.

THE following delegates from the Medical Association of Georgia to the American Medical Association left Atlanta last week for New Orleans, the place of meeting: Dr. Henry F. Campbell, Augusta, President of the American Association; Dr. E. C. Goodrich, Augusta, Treasurer of the State Association; Dr. J. B. Roberts, Sandersville; Dr. J. W. Flanders, Wrightsville; Dr. E. H. Richardson, Cedartown; Dr. Hightower, Dublin; Dr. J. C. Solomon, Perry; Dr. T. O. Powell, Milledgeville; Dr. E. L. Connally and W. A. Love, Atlanta.

A DOUBLE TONGUE.—The following peculiar case is furnished us by Dr. F. M. Brantley, of Senoia, Ga.:

"Mrs. U. has two tongues—one upon the dosum of the other, the upper one being only a little shorter and narrower, both having frenums, and seem to offer no impediment to speech or deglutition. I cannot say that this is congenital; being the first to discover this aberration after she was grown and married. I watched this case for over thirty years, which is still in *statu quo*. I do not remember of ever having read of a similar case."

THE DEADLY TEAPOT.—"While good temperance people are decrying liquor," says a leading American physician, "they seldom stop to think how much harm is being done by an abuse of a beverage to which so many of them are devoted. I just came from attending a case of a five-year-old babe who is ruined for life by its parents indulging in tea-drinking. The child became very nervous and dyspeptic, and they sent for me. I asked them how much tea the child drank. 'About two cups at each meal, and several between meals,' was the reply. "You see," the physician continued, "they let the teapot stand on the stove all day. Thus the tannic acid is extracted, which serves to turn the linings of the stomach into leather, and brings on dyspepsia and kindred diseases. Yes, you will find hundreds of women, young girls, and aged women, and occasionally a man, who have completely ruined their nervous system by the excessive use of common tea. It would be a blessing to mankind when a temperance crusade can spare wind enough from its attack on alcohol to assail tea."—*British Med. Jour.*

METEOROLOGICAL summary for March at this station:

| | |
|---|---|
| Highest temperature, March 31, | 69.5 |
| Lowest temperature, March 23. | 20.4 |
| Range, | 49.1 |
| Greatest daily range of temperature, March 17, | 27.6 |
| Least daily range of temperature, March 4, | 9.0 |
| Mean daily range of temperature, | 18.1 |
| Mean daily dew-point, | 30.4 |
| Mean daily relative humidity, | 56.0 |
| Prevailing direction of wind, | W. |
| Total movement of wind, | 7,494 miles. |
| Highest velocity of wind and direction, | 32 miles—S.W. |
| No. of foggy days, | none. |
| No. of clear days, | 11 |
| No. of fair days, | 13 |
| No. of cloudy days, | 7 |
| No. of days on which rain or snow fell, | 10 |

THE Medical Association of the State of Alabama held its thirty-seventh annual session at Greenville, April 14–17, Dr. B. W. Riggs, President, in the chair. There were over one hundred members present. The following are the officers for the ensuing year:

*President.*—Dr. F. M. Peterson, of Greensboro.

*Vice-Presidents.*—Dr. Star, of Wilcox county, and Dr. Richard M. Fletcher, of Madison county.

*State Board of Censors.*—Drs. Riggs, Dement and Ketchum.

*Councillors.*—Drs. Herbert, Goodwin, Bragg, J. B. Kendrick, Norris, Wheelan, Nicholson, Lowry, and Inge.

*Members of the State Board of Health.*—Drs. Riggs, Ketchum and Dement.

*Orator.*—Dr. Bragg, of Lowndes.

The Association will meet at Anniston next year.

A CASE of alleged maternal impression is recorded by a correspondent of the *British Medical Journal*, March 28, 1885, occurring in a woman, aged forty-two, multipara, whose menstruation stopped in March. During the same month she saw the "Two-headed Nightingale," fainted at the sight, and was so affected that three days after she attempted suicide by jumping from a window. In November she expressed to a medical man, who suspected the existence of twins, a hope that they would not be "Siamese." In January she gave birth to a twin monstrosity, the two children being connected from neck to umbilicus. Both were dead.—*Boston Medical and Surgical Journal.*

MARRIAGE OF MRS. TOM THUMB.—The widow of Tom Thumb was married, April 6, to Count Primo Magri, an Italian dwarf of about her own height, at the Church of the Holy Trinity, New York. Not long since a fat woman, weighing, according to the announcement on the bill-boards, 596 pounds, was married to a professional "Albino" at a dime museum on the Bowery; and when the ceremony was completed the band, not inappropriately, struck up the air, "What shall the harvest be?"—*Gaillard's Medical Journal.*

PREGNANCY WITH PERSISTENT HYMEN.—Arnoux (London *Medical Record*) narrates the case of a young married woman, aged 23, who sought advice for constant vomiting. She doubted that she could be pregnant, as an obstacle existed which prevented perfect connection. On examination the finger was arrested at about an inch and a third from the ordinary site of the vulvo-vaginal orifice by a diaphragm, which in the upper right part with difficulty permitted the entrance of the tip of the finger; beyond this could be felt another membrane, through which the finger could not be passed. With the uterine sound a very small aperture was found in the upper part of the posterior fold. The apertures did not correspond, that in the upper fold being covered by the anterior lower fold. Pregnancy was made out by rectal examination. The membranes were divided by a probe-pointed bistoury, and the woman was discharged in a few days. This case is interesting from the occurrence of pregnancy with a persistent hymen, and from the unusual site of the hymen.—*Med. Review.*

" MELLIN'S FOOD FOR INFANTS AND INVALIDS.—A recent analysis by Mr. G. W. Wigner, the president of the Society of Public Analysis of England, throws considerable light, not only on the composition, but on the physiological action of this popular preparation. It appears that it contains nearly 87 per cent. of dextrine, maltose, etc., soluble in cold water.

" As Mr. Wigner points out, it is not a mere starch or sugar food, but a soluble preparation, containing those nitrogenous and phosphatic principles which contribute largely to the growth of bone and tissue in young children. Being thoroughly malted, it is not only readily digestible itself, but actually assists in the digestion of milk and other foods with which it is mixed. It must of necessity be of great value in the case of feeble infants who cannot digest ordinary starchy foods. Mr. Wigner's analysis has evidently been performed with great care, and is of much interest".—*British Medical Journal,*

THE CHEAPNESS OF QUININE.—Quinine is said to be cheaper almost than it has ever been. Few facts are more important to mankind, next to the price of wheat and meat. In our temperate climate, the price of quinine is of less interest than in hot climates and marshy regions. Yet with us how large is the scale of its use, and how specific the benefits which it confers in neuralgia, in puerperal and other pyrexiæ, in rheumatic fever, and in debility of all kinds. But in tropical countries, and especially for Europeans in these, it is almost essential to the preservation of life. In these days of Suez and Panama canals, of African exploration and colonization, and of Nile expeditions, the cheapness of quinine is of pleasant and prodigious consequence. It would be very instructive if there were attached to all these undertakings a subordinate of the Registrar-General. We should then know on what a scale life is sacrificed where quinine is withheld, and how unequal is the risk to the rich man who has the quinine and the poor man who has it not.—*Lancet.*

AMERICAN SURGICAL ASSOCIATION.—The meeting of the Association was held in the Army Medical Museum, Washington, April 21, 22, 23 and 24, 1885. The following papers were presented: "The Field and Limitation of the Operative Surgery of the Human Brain," by John B. Roberts, M. D., Philadelphia; "An Experimental and Clinical Study of Air Embolism," by N. Senn, M. D., Milwaukee, Wis.; "Nephrectomy: Its Indications and Contra-indications," by Samuel W. Gross, M. D., Philadelphia; "Nepho-lithotomy," by L. McLane Tiffany, M. D., Baltimore; "The Healing Arteries in Man and Animals after Ligature," by J. Collins Warren, M. D.; "The Immediate Cure of Fistula in Ano," by Stephen Smith, M. D., New York; "Etiology of Tetanus," by P. S. Connor, M. D., Cincinnati; "Some Points in the Surgery of the Hypertrophied Prostate," by J. W. Gouley, M. D., New York; "Phosphorus Necrosis: Its Causes, Treatment, and Prevention, with Reports of Cases," by J. Ewing Mears, M. D., Philadelphia.

WE are indebted to Dr. John H. Rauch, secretary of the Illinois State Board of Health, for the biennial message of the Governor of Illinois to the thirty-fourth General Assembly, 1885. From it we extract the following on the State Board of Health: "The State Board of Health, which was in its inception very difficult to establish by legislative enactment, has steadily grown in usefulness and popular favor, until now it is one of the most important bureaus of the State government. By reason of the able management of its members, and especially of its secretary, the medical profession of the State has been very much elevated and improved. Incompetent beginners have been prevented from practicing. The grade of medical education required for practice has been raised to a respectable and safe standard, while mountebanks and quacks have been driven from the practice of their wiles and deceptions on the people of this State. The health of the citizens and their protection from inroads of contagious and epidemic diseases have been faithfully and carefully watched. Rules for sanitary care and regulation and instruction as to prevention and cure of prevalent and especially dangerous diseases, have also been so successfully published and promulgated that it is believed thousands of lives have been saved."

PUCK'S "REALISTIC RECIPE" FOR HOMŒPATHIC MEDICINE.—

A grain of medicine you take
And drop it in Superior Lake;
Mix it and stir it thoroughly,
Then of the mixture in the sea
Put just one drop and stir it well,
So neither taste nor touch nor smell
Of medicine within is found;
Then take of sugar just a pound,
And medicate it with one drop
Of the aforesaid mingled slop;
Each day three times take half a grain,
Till you are dead or free from pain.

This is about as bad as the advice of the homœopathic physician in a play that was acted sometime since at Wallack's, who gave his patient a little phial and told him to put five drops of its contents in a quart of water, and take a teaspoonful every Wednesday night.—*Gaillard's Medical Journal.*

DR. J. P. TAYLOR and Dr. F. M. Brantley, of Senoia, and Dr. R. H. Taylor, of Griffin, were in the city last week.

CONCENTRATED FOODS.—Medical men are now recognizing the value of malt extracts as foods in cases of deficient assimilation. That their use is extending may be taken for granted by the number of exhibitors of concentrated foods in the exhibition at South Kensington last year. Important improvements have recently been made in the manufacture of malt extracts, which are now prescribed in a variety of forms. One of the most effective combinations in dyspepsia, cholera infantum, and all diseases resulting from imperfect nutrition, is Maltine with pepsine and pancreatine, containing, as it does, three of the all-important digestive agents, diastase being one of the constituents of Maltine. Dyspepsia in most cases will be found to yield to the medicinal properties of this combination, while the system is invigorated by its nutritive qualities. It will be found a useful remedy also for constipation and chronic diarrhœa resulting from mal-nutrition. Not only is Maltine of itself of great value in certain cases, but it may be combined with the most valuable alteratives known—such as iodides, bromides and chlorides—and is found to be a remedy of high value in all depraved conditions of the blood. The Maltine manufactured by the Maltine Manufacturing Company of New York bears a high name, and this has been still further emphasized by the award of the gold medal of the Health Exhibition, London, for their malt extract known as Maltine (malted wheat, barley and oats), the only preparation composed of these three cereals. Professor Charles R. C. Tichborne, after an examination of the principal unfermented extracts of malt in the market, finds that Maltine is the richest in two of the most important ingredients in these foods—namely, the phosphates or bone-formers, and that peculiar farinaceous digestive agent called diastase. Maltine may be said to consist of about eighty per cent. of pure food in its most concentrated and assimilable form. This eighty per cent. may be divided as follows: five and a half per cent. of flesh-formers; seven per cent. of heat-givers; two per cent. of bone-formers; add to this the diastase, which imparts to it the curious power of digesting all farinaceous food outside itself, and we have in Maltine a most valuable adjunct to our invalid diet. In respect to the diastase, Maltine seems remarkably energetic, and at the temperature of the human body one part liquified twenty parts of starch in two minutes, and had completely changed or digested that body in about an hour. Maltine possesses all the characteristics of a cereal extract as prepared from the grain, and there can be no question about the genuineness of this preparation. It is only necessary to consult any work upon dietetics to see that there is considerable difference in the composition of the various grain crops. By combining these three important substances—barley, oats and wheat—a food is obtained which represents the average composition of the three cereals, and that food already digested for use, a condition of immense value to the physician in those special cases where the digestive functions are impaired.—*Midland Medical Journal*, *London*.

